# The Effect of Muscle Direction on the Predictions of Finite Element Model of Human Lumbar Spine

**DOI:** 10.1155/2018/4517471

**Published:** 2018-01-03

**Authors:** Rui Zhu, Wen-xin Niu, Zhi-peng Wang, Xiao-long Pei, Bin He, Zhi-li Zeng, Li-ming Cheng

**Affiliations:** ^1^Spine Division of Orthopaedic Department, Tongji Hospital, Tongji University School of Medicine, 389 Xincun Road, Shanghai 200065, China; ^2^School of Electronics and Information Engineering, Tongji University, Shanghai 201804, China

## Abstract

The normal physiological loads from muscles experienced by the spine are largely unknown due to a lack of data. The aim of this study is to investigate the effects of varying muscle directions on the outcomes predicted from finite element models of human lumbar spine. A nonlinear finite element model of L3–L5 was employed. The force of the erector spinae muscle, the force of the rectus abdominis muscle, follower loads, and upper body weight were applied. The model was fixed in a neural standing position and the direction of the force of the erector spinae muscle and rectus abdominis muscle was varied in three directions. The intradiscal pressure, reaction moments, and intervertebral rotations were calculated. The intradiscal pressure of L4-L5 was 0.56–0.57 MPa, which agrees with the* in vivo* pressure of 0.5 MPa from the literatures. The models with the erector spinae muscle loaded in anterior-oblique direction showed the smallest reaction moments (less than 0.6 Nm) and intervertebral rotations of L3-L4 and L4-L5 (less than 0.2 degrees). In comparison with loading in the vertical direction and posterior-oblique direction, the erector spinae muscle loaded in the anterior-oblique direction required lower external force or moment to keep the lumbar spine in the neutral position.

## 1. Introduction

Finite element (FE) analysis has been used extensively as a powerful computational tool for resolving difficult or complex clinical and biomechanical situations [1]. The quality of the outcome is strongly influenced by the anatomical geometry [2], material properties [1], and boundary and loading conditions [3]. The anatomical geometry is typically generated from medical images of a representative subject and the material properties are often measured from cadaveric specimens. However, given the highly invasive nature of recording* in vivo* data, the normal physiological loading in the spine is still largely unknown due to a lack of data on muscle forces [4].

Various assumptions have been made in experimental and numerical studies. Compressive force or/and pure moment was used earlier [4]. Recently, the concept of using a single compressive follower load was introduced to represent body weight and muscles [3, 5, 6]. The follower load is a compressive load applied along a follower load path that approximates a tangent to the curve of the lumbar spine. For posture, the musculature is one of the most important components for preserving spinal stability [7, 8]. Many studies have attempted to incorporate muscle forces into FE simulations in order to improve the accuracy of the model. Kong and Goel [9] developed an optimization-based force model with experimental input to predict muscle forces in a single-joint FE model. Some authors estimated muscle forces using a kinematics-driven algorithm [10, 11] and an EMG-assisted optimization method [10, 12]. Calisse et al. [13] and Zander et al. [14] recorded* in vivo *data from internal spinal fixators to predict muscle forces. Khurelbaatar et al. [15] developed a spinal FE model with trunk muscles and estimated muscle forces using an optimization technique.

However, it was difficult to share the above-mentioned muscle forces for other researchers due to their private optimization techniques. We have previously carried out sensitivity studies when transferring muscle forces from a shared inverse static model to load an FE model [16, 17]. The findings showed that many parameters have to be considered when combining two biomechanical models and artificial errors may be induced in the model which may have a significant impact on the outcome.

Rohlmann et al. [26] considered four groups of loads: the force of the erector spinae (ES) muscle, the force of the rectus abdominis (RA) muscle, follower loads, and upper body weight. The magnitude of loads and action points were estimated from representative studies and data, but all muscle forces were applied in a vertical direction. This is an oversimplification of the true loading condition in the body. In our opinion, more realistic muscle directions may lead to more physiological loads. FE analysis regarding mechanism for some diseases and biomechanical efficacy for surgery may be beneficial. Therefore, the aim of this study is to investigate the effects of muscle direction on the simulation outcomes during FE modeling of lumbar spine.

## 2. Materials and Methods

### 2.1. Finite Element Model of L3–L5

A nonlinear FE model of L3–L5 [27] was employed for this study. The vertebral bodies of the lumbar spine were modeled as isotropic cortical shells with a thickness of 0.5 mm [28, 29], also with a transverse isotropic cancellous core and isotropic posterior bony structures. Cartilaginous endplates were assigned a thickness of 0.8 mm [30, 31]. The intervertebral discs were comprised of an incompressible nucleus pulposus surrounded by a composite annulus fibrosus. The annuli fibrosi were modeled as fiber-reinforced hyperelastic composites. Rebar elements of two times seven layers were used to represent the fibers and the stiffness was increased as the fibers radiated out from the center [21]. A 0.25 mm thick cartilaginous layer was also added to each facet articular surface, and a gap of 0.5 mm was created between the curved facet joints [23]. All seven ligaments of the lumbar spine were represented by tension-only spring elements with nonlinear material properties [20, 25]. The material properties of the various tissues in the FE model are listed in Table 1.

### 2.2. Validation of Mobility

In order to validate the mobility of the model, 7.5 Nm moments were applied to the top of L3 to simulate flexion, extension, right lateral bending, and right axial rotation, while all six degrees of freedom of the inferior endplate of L5 were rigidly fixed. The intervertebral rotation (IVR) of L3-L4 and L4-L5 was calculated and compared with* in vitro* experiment data [18].

### 2.3. Loading and Evaluation

**Figure 1:**
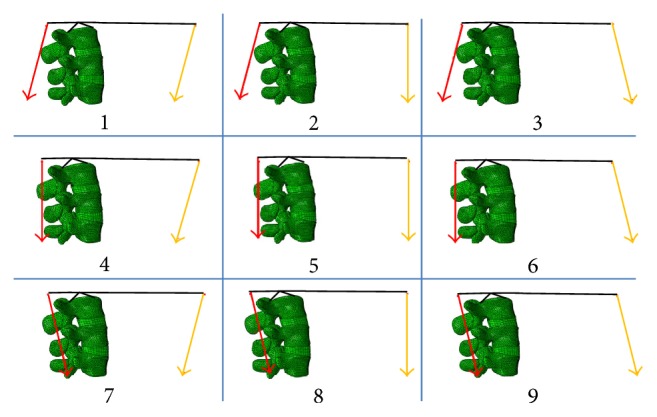
Nine loading conditions simulated by varying the direction of the erector spinae (ES) muscle (red line) and rectus abdominis (RA) muscle (yellow line). The muscle forces were assigned to act in the posterior-oblique direction, vertical direction, and anterior-oblique direction.

Four groups of loads [26] were applied to the models in this study. The global back muscle forces were represented by the force of ES with a magnitude of 170 N acting 40 mm dorsal to the related disc center. Similarly, the force of RA was used to represent the global abdominal muscle forces. The magnitude was set at 20 N and acted 153 mm ventral to the related disc center. The action line of ES and RA was varied in three directions: anterior-oblique direction, vertical direction, and posterior-oblique direction. The angles between the anterior-oblique direction and vertical direction and between the posterior-oblique direction and vertical direction were 15 degrees. Nine loading conditions were simulated by varying the direction of action of ES and RA (Figure 1).

Local muscle forces were represented by a follower load of 200 N which acted along the curvature of the spine. A representative upper body weight of 260 N acted 30 mm ventral to the center of the upper disc. The path of the follower load and the upper body weight were constant in all loading conditions. The inferior endplate of L5 was rigidly fixed in six degrees of freedom.

The intradiscal pressure (IDP), reaction moments, and intervertebral rotations (IVRs) were calculated. The FE program ABAQUS 6.13 (Dassault Systèmes, Versailles, France) was used for all simulations.

## 3. Results

### 3.1. Validation

**Figure 2:**
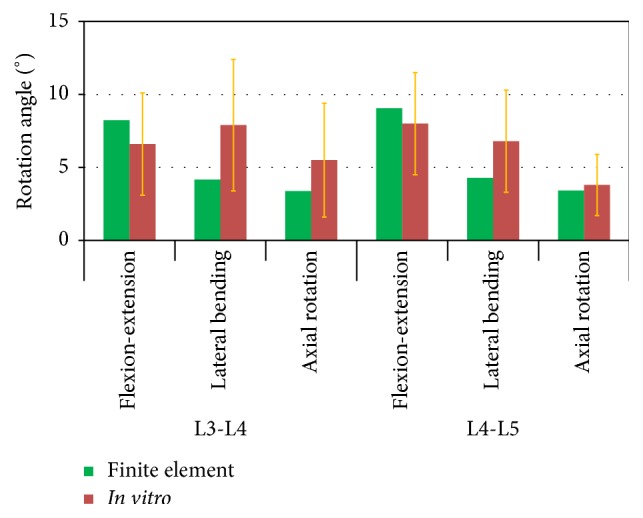
Comparison of the calculated intervertebral rotations (IVRs) of L3-L4 and L4-L5 to experimental data [18] for different loading conditions. The standard deviations of the* in vitro* results are also given.

The IVRs were recorded after applying moments of 7.5 Nm in flexion-extension, lateral bending, and axial rotation. The results for L3-L4 and L4-L5 were within the range of* in vitro *experimental data [18] (Figure 2).

### 3.2. Intradiscal Pressure (IDP)

The IDP recorded for L4-L5 was within 0.56–0.57 MPa for all nine loading conditions, which was similar to the 0.5 MPa* in vivo* pressure reported by Wilke et al. [32]. Therefore, a truly physiological IDP was achieved by the combination of the axial spinal load and upper body weight. Varying the direction of the muscle forces only had a minor influence on IDP for all nine loading conditions.

### 3.3. Reaction Moment

**Figure 3:**
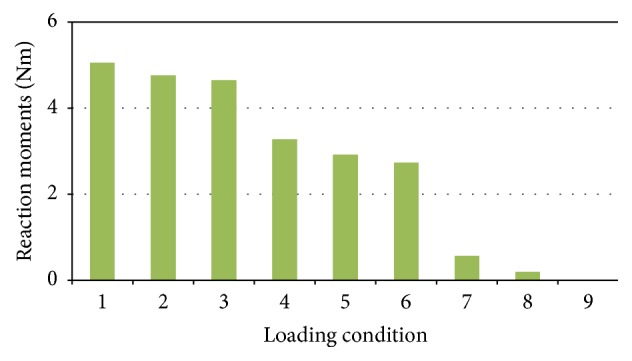
Reaction moments against boundary condition for the nine different loading conditions.

Reaction moments acting against the boundary conditions which maintained the FE model in a neutral position were calculated and compared among the nine models (Figure 3). The three models (loading condition 7–9) with ES acting in an anterior-oblique direction showed smaller reaction moments. Overall, loading condition 9 produced the smallest reaction moment, while loading condition 1 produced the greatest. A greater reaction moment can be interpreted to mean that a greater external load is required to poise muscle forces and upper body weight.

### 3.4. Intervertebral Rotation

**Figure 4:**
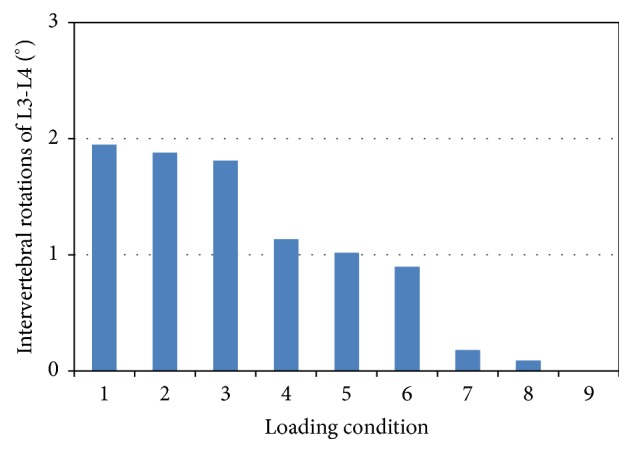
Calculated intervertebral rotations of L3-L4 functional unit.

**Figure 5:**
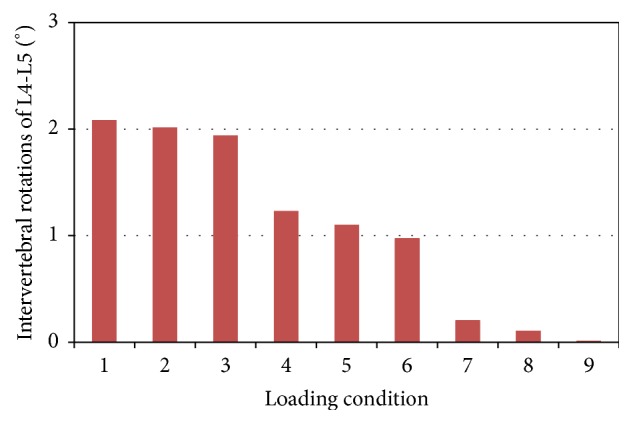
Calculated intervertebral rotations of L4-L5 functional unit.

In these loading conditions, the L3-L4 spinal functional unit showed flexion, while the L4-L5 showed extension. In loading condition 1–3, the combination of ES in the posterior-oblique direction and RA in three different directions resulted in large IVRs of L3-L4 and L4-L5 up to 2 degrees (Figures 4 and 5). In these three loading conditions where the force of ES was applied in the vertical direction, the IVRs of L3-L4 and L4-L5 had medium values. The smallest IVRs occurred in the loading conditions with ES in the anterior-oblique direction.

## 4. Discussion

The musculature plays an important role in maintaining the stability of the spine, but muscle forces are often difficult to measure due to the invasive methods required for* in vivo* measurements. Attempts have been made to incorporate muscle forces into FE models [9–15, 26, 33], but most of these forces were estimated based on locally developed algorithm/data. A generally practical method can be helpful for applications of muscle forces and driving private FE models. Muscle forces combined with follower loads and upper body weight investigated by Rohlmann et al. [26] may play an important role. A nonlinear FE model was employed in the present study in order to investigate how the direction of application of muscle forces impacts the motion of the lumbar spine. The magnitudes of all forces as well as the direction of the follower loads and upper body weight were kept constant. The physiological conditions are variable, so a sensitivity study was carried on. The action line of the ES and RA muscle forces was varied in three directions: anterior-oblique direction, vertical direction, and posterior-oblique direction. In total, nine loading conditions were simulated through a combination of the different directions of ES and RA. Motion and loading data was recorded for further analysis and to determine the impact of muscle direction on the spinal motion segment.

The model was validated by confirming that the IVRs of the L3-L4 and L4-L5 spinal functional units were within the range of* in vitro* experimental data [18]. There were large variations for intervertebral rotations of the human body. Thus, the FE predictions in our study represent a part of populations instead of all. In addition, the calculated IDP of L4-L5 was 0.56–0.57 MPa, which was similar to the 0.5 MPa value reported in an* in vivo* study by Wilke et al. [32]. Thus, the spinal compressive loads in this study created by a combination of muscle forces and upper body weight are in line with physiological loads experienced under similar conditions, at least with quite a part of populations, since there are large variations in human bodies. Varying the direction of the muscle forces only had a minor influence on IDP when the spinal position is unchanged.

As the IDP and IVR are sensitive to posture [32], the parameters calculated for nine loading conditions cannot be compared in a unified way without restriction. Therefore, this study rigidly fixed L3 in a neutral standing position. The reaction moments to the boundary conditions reflect the degree of deviation to the neutral standing position. A greater reaction moment means a greater external load is needed to poise the muscle forces and upper body weight. The magnitudes of the reaction moments in loading conditions 7, 8, and 9 were relatively small (Figure 3). ES acting in an anterior-oblique direction needed a lower external force or moment to keep the spinal FE model in a neutral position, in comparison to when acting in a vertical direction or posterior-oblique direction. This finding was in agreement with anatomical observations and other biomechanical models [34]. The direction of RA also affected the reaction moment but had less of an influence than ES. The biomechanical environment of the abdomen is more complicated than shown in this model, because both of abdominal muscles and abdominal pressure take part in maintaining stability.

In physiological environment, upper body weight and muscle forces are well balanced. Due to the L3 being fixed in the FE model, the overall IVR was 0 degrees. However, the IVR for each spinal motion unit did vary with different loads. In Figures 4 and 5, the loading conditions with ES acting in an anterior-oblique direction showed smaller IVRs for L3-L4 and L4-L5. A satisfied equilibrium was achieved by the back muscle, abdominal muscle, follower load, and body weight. This is in accordance with the one from reaction moment. Similarly, the variations caused by varying the direction of RA were rather small.

There are some limitations in this study that should be noted. Firstly, the geometry and material properties were constant across all models. This may not be representative of the entire population. Secondly, only two spinal functional units were involved and only effect in sagittal direction was investigated. Thirdly, the effect of local muscles was simulated by a follower load of 200 N, which is a compressive load applied along a follower load path, thus minimizing the shear forces. A more detailed model including additional muscles would be beneficial in further studies. Although several simplifications were made, the results drawn from the comparison among the loading conditions are still valid since the direction of the muscle forces was changed in a parameterized way and the configurations were the same for all loading conditions. Thus, the results of this study can be viewed as a comparative analysis that provides information for applying muscle forces.

## 5. Conclusion

The spinal compressive loads delivered by muscle forces and upper body weight in this study are comparable to physiological conditions. This study also found that the muscle forces and upper body weight are capable of maintaining equilibrium in the case of erector spinae muscle in anterior-oblique direction. The erector spinae muscle acting in the anterior-oblique direction needed a lower external force or moment to keep the spinal FE model in a neutral position, in comparison to the vertical direction and posterior-oblique direction.

## Figures and Tables

**Table 1:** Material properties of tissues in the finite element model.

Component	Material properties	References
Cancellous bone (transverse isotropic)	*E* = 200/140 MPa, *υ* = 0.45/0.315 (axial/radial)	[19]
Cortical bone	*E* = 10,000 MPa, *υ* = 0.30	[20]
Posterior bony elements	*E* = 3,500 MPa, *υ* = 0.25	[21]
Nucleus pulposus	Incompressible	[20]
Ground substance of annulus fibrosis	Hyperelastic, neo-Hookean, *C*_10_ = 0.3448, *D*_1_ = 0.3	[22]
Cartilage of facet joint	Soft contact	[23, 24]
Ligaments	Nonlinear	[20, 25]

## References

[1] Dreischarf M., Zander T., Shirazi-Adl A. (2014). Comparison of eight published static finite element models of the intact lumbar spine: Predictive power of models improves when combined together. *Journal of Biomechanics*.

[2] Bredbenner T. L., Eliason T. D., Francis W. L., McFarland J. M., Merkle A. C., Nicolella D. P. (2015). Development and validation of a statistical shape modeling-based finite element model of the cervical spine under low-level multiple direction loading conditions. *Frontiers in Bioengineering and Biotechnology*.

[3] Rohlmann A., Zander T., Rao M., Bergmann G. (2009). Realistic loading conditions for upper body bending. *Journal of Biomechanics*.

[4] Zhu R., Yu Y., Zeng Z. L., Cheng L. M. (2015). A Review of the Static Loads Applying on the Finite Element Models of the Lumbar Spine. *Journal of Medical Imaging Health Informatics*.

[5] Naserkhaki S., El-Rich M. (2017). Sensitivity of lumbar spine response to follower load and flexion moment: finite element study. *Computer Methods in Biomechanics and Biomedical Engineering*.

[6] Rohlmann A., Zander T., Rao M., Bergmann G. (2009). Applying a follower load delivers realistic results for simulating standing. *Journal of Biomechanics*.

[7] Vazirian M., Shojaei I., Tromp R. L., Nussbaum M. A., Bazrgari B. (2016). Age-related differences in trunk intrinsic stiffness. *Journal of Biomechanics*.

[8] Shahvarpour A., Shirazi-Adl A., Mecheri H., Larivière C. (2014). Trunk response to sudden forward perturbations - Effects of preload and sudden load magnitudes, posture and abdominal antagonistic activation. *Journal of Electromyography & Kinesiology*.

[9] Kong W. Z., Goel V. K. (2003). Ability of the finite element models to predict response of the human spine to sinusoidal vertical vibration. *The Spine Journal*.

[10] Arjmand N., Gagnon D., Plamondon A., Shirazi-Adl A., Larivière C. (2009). Comparison of trunk muscle forces and spinal loads estimated by two biomechanical models. *Clinical Biomechanics*.

[11] Ghezelbash F., Shirazi-Adl A., Arjmand N., El-Ouaaid Z., Plamondon A., Meakin J. R. (2016). Effects of sex, age, body height and body weight on spinal loads: Sensitivity analyses in a subject-specific trunk musculoskeletal model. *Journal of Biomechanics*.

[12] Mohammadi Y., Arjmand N., Shirazi-Adl A. (2015). Comparison of trunk muscle forces, spinal loads and stability estimated by one stability- and three EMG-assisted optimization approaches. *Medical Engineering & Physics*.

[13] Calisse J., Rohlmann A., Bergmann G. (1999). Estimation of trunk muscle forces using the finite element method and in vivo loads measured by telemeterized internal spinal fixation devices. *Journal of Biomechanics*.

[14] Zander T., Rohlmann A., Calisse J., Bergmann G. (2001). Estimation of muscle forces in the lumbar spine during upper-body inclination. *Clinical Biomechanics*.

[15] Khurelbaatar T., Kim K., Kim Y. H. (2015). A cervico-thoraco-lumbar multibody dynamic model for the estimation of joint loads and muscle forces. *Journal of Biomechanical Engineering*.

[16] Zhu R., Zander T., Dreischarf M., Duda G. N., Rohlmann A., Schmidt H. (2013). Considerations when loading spinal finite element models with predicted muscle forces from inverse static analyses. *Journal of Biomechanics*.

[17] Zhu R., Rohlmann A. (2014). Discrepancies in anthropometric parameters between different models affect intervertebral rotations when loading finite element models with muscle forces from inverse static analyses. *Biomedizinische Technik. Biomedical Engineering*.

[18] Ilharreborde B., Shaw M. N., Berglund L. J., Zhao K. D., Gay R. E., An K. (2011). Biomechanical evaluation of posterior lumbar dynamic stabilization: an in vitro comparison between Universal Clamp and Wallis systems. *European Spine Journal*.

[19] Ueno K., Liu Y. K. (1987). Three-dimensional nonlinear finite element model of lumbar intervertebral joint in torsion. *Journal of Biomechanical Engineering*.

[20] Rohlmann A., Zander T., Schmidt H., Wilke H.-J., Bergmann G. (2006). Analysis of the influence of disc degeneration on the mechanical behaviour of a lumbar motion segment using the finite element method. *Journal of Biomechanics*.

[21] Shirazi-Adl A., Ahmed A. M. (1986). Axial torque alone and combined with compression. *The Spine Journal*.

[22] Eberlein R., Holzapfel G. A., Schulze-Bauer C. A. J. (2001). An anisotropic model for annulus tissue and enhanced finite element analyses of intact lumbar disc bodies. *Computer Methods in Biomechanics and Biomedical Engineering*.

[23] Goel V. K., Kim Y. E., Lim T.-H., Weinstein J. N. (1988). An analytical investigation of the mechanics of spinal instrumentation. *The Spine Journal*.

[24] Sharma M., Langrana N. A., Rodriguez J. (1995). Role of ligaments and facets in lumbar spinal stability. *The Spine Journal*.

[25] Nolte L. P., Panjiabi M. M., Oxland T. R. (1990). Biomechanical properties of lumbar spinal ligaments. *Clinical Implant Materials, Advancesin Biomaterials*.

[26] Rohlmann A., Bauer L., Zander T., Bergmann G., Wilke H.-J. (2006). Determination of trunk muscle forces for flexion and extension by using a validated finite element model of the lumbar spine and measured in vivo data. *Journal of Biomechanics*.

[27] Zeng Z., Zhu R., Wu Y. (2017). Effect of Graded Facetectomy on Lumbar Biomechanics. *Journal of Healthcare Engineering*.

[28] Thomas Edwards W., Zheng Y., Ferrara L. A., Yuan H. A. (2001). Structural features and thickness of the vertebral cortex in the thoracolumbar spine. *The Spine Journal*.

[29] Zhu R., Cheng L.-M., Yu Y., Zander T., Chen B., Rohlmann A. (2012). Comparison of four reconstruction methods after total sacrectomy: A finite element study. *Clinical Biomechanics*.

[30] Ayturk U. M., Puttlitz C. M. (2011). Parametric convergence sensitivity and validation of a finite element model of the human lumbar spine. *Computer Methods in Biomechanics and Biomedical Engineering*.

[31] Womack W., Woldtvedt D., Puttlitz C. M. (2008). Lower cervical spine facet cartilage thickness mapping. *Osteoarthritis and Cartilage*.

[32] Wilke H.-J., Neef P., Hinz B., Seidel H., Claes L. (2001). Intradiscal pressure together with anthropometric data—a data set for the validation of models. *Clinical Biomechanics*.

[33] Zhu R., Niu W.-X., Zeng Z.-L. (2017). The effects of muscle weakness on degenerative spondylolisthesis: A finite element study. *Clinical Biomechanics*.

[34] Putzer M., Ehrlich I., Rasmussen J., Gebbeken N., Dendorfer S. (2016). Sensitivity of lumbar spine loading to anatomical parameters. *Journal of Biomechanics*.

